# From automation to augmentation: a retrieval-augmented large language model framework for reshaping learning experiences in higher education

**DOI:** 10.3389/fpsyg.2026.1870870

**Published:** 2026-06-16

**Authors:** Wang Lingling, Sun Shijie, Liu Xueyi

**Affiliations:** 1 Jiangsu Medical College, Yancheng, Jiangsu, China; 2 Nanjing Medical University, Nanjing, Jiangsu, China

**Keywords:** behavioural analytics, educational chatbot, higher education, human and AI collaboration, large language models, learning analytics, retrieval-augmented generation

## Abstract

Generative artificial intelligence is now embedded in everyday university work, yet most evidence on its educational value still comes from surveys of student perception rather than from what students do. We reanalyzed the public log of *Prof. Leodar*, a course-specific retrieval-augmented chatbot deployed in an undergraduate data science module at Nanyang Technological University, Singapore. The original deployment study collected survey responses from 34 of 154 enrolled students (a 22% response rate). It released the complete query log of 12,330 anonymized learner queries over a 14-week pilot, in which the chatbot was powered by Anthropic Claude 3 (Sonnet) and a FAISS vector store. Because the survey sample is small and self-selected, and because the query log is a convenience sample clustered at the student level, this is a descriptive analysis: we report effect sizes and predictive accuracy as within-corpus summaries and do not generalize to other cohorts. We propose a query-level *Automation-to-Augmentation* framework that separates interactions in which the system simply delivers an answer from interactions that scaffold reasoning, debugging, or interpretation. Each query is assigned a topical category, one of five retrieval-use types, an augmentation label, and a *Learning Augmentation Index* (LAI) on a 0–100 scale; reading complexity is captured using the Flesch–Kincaid grade-level measure. We summarized the corpus with frequencies, Cramer’s V (reported descriptively, with Bonferroni-corrected *p*-values), Kruskal–Wallis comparisons, and a 5-fold cross-validated logistic regression. Augmentation-oriented behavior accounts for 46.6% of all queries. The four technical categories (programming and debugging, data visualization, machine learning and modeling, and statistics and mathematics) together account for 57.3% of the corpus and carry the highest LAI (58.3 to 61.1). Retrieval-use type is more strongly associated with augmentation than topical category (Cramer’s V = 0.226 versus 0.166), and the classifier recovers augmentation labels at AUC = 0.792 (accuracy 0.745, F1 0.666). Because the labels and the LAI are rule-based and the dataset contains no learning-outcome information, the contribution is best read as a transparent, reproducible behavioral analytics framework for course-specific RAG, rather than a measurement of learning itself.

## 1 Introduction

Generative artificial intelligence has become a normal part of university studies. Students use language models to request explanations, coding help, writing feedback, revision tips, and assessment preparation (Kasneci et al., 2023). The same system that supports deep understanding can also produce a finished answer that the student submits without engaging with it (Tlili et al., 2023). This is the tension every instructor now lives with, and it shapes the question we ask in this study.

Most published research evaluates AI in education either at the system level (does the model give correct answers?) or at the perception level (do students say it helped?) (Chan and Hu, 2023; Albadarin et al., 2024; Saude et al., 2024). Both are useful, but neither tells us what a specific interaction did for a specific learner. A query that asks “what does standard deviation mean?” and a query that asks “give me the answer to question 4” both count as one interaction and both can return a correct response, yet only the first is plausibly a moment of learning.

Retrieval-augmented generation (RAG) (Lewis et al., 2020) is a natural fit for university teaching because most student questions depend on local material such as these notes, this assessment, and this week’s tutorial (Li et al., 2025; Swacha and Gracel, 2025). The deployment we reanalyze is described by Thway et al. (2025). Prof. Leodar developed a course-specific chatbot for an undergraduate data science module at Nanyang Technological University. It retrieves from the lecturer’s own course materials before generating a reply, and was offered to students over a 14-week semester. At the time of data collection by Anthropic Claude 3 (Sonnet) on AWS Bedrock, it was powered with a FAISS vector store over OpenAI embeddings. Of the 154 enrolled students, 34 (22%) consented to participate in the accompanying survey, and all interaction logs were anonymized at source. The full query log and the underlying production code were released publicly as supplementary material to (Thway et al., 2025) on Mendeley Data (DOI: 10.17632/9mrxwtfh6w.1), as we analyzed here. Claude 3 (Sonnet) is no longer the current model in the Claude family, and interaction patterns observed with one specific model and one specific cohort cannot be assumed to transfer to other large language models or other courses.

Our argument is simple. If we want to understand what a course-level RAG deployment is doing in observable behavioral terms, we should look at the queries themselves. We define a query as augmentation-oriented when it asks the system to support understanding, diagnosis, interpretation, or guided practice, and automation-oriented when it asks for a finished output with no learning context attached. This distinction is observable in the surface text of the query, which means it can be measured at scale. The distinction is a property of what the learner *asks for*, not of what the learner *learns*. The public dataset contains no assessment data, no dialogue history, and no measure of conceptual gain, so the framework cannot support claims that augmentation-oriented queries produce better learning outcomes. It is a behavioral analytics scheme that classifies the surface-level form of student-chatbot interaction and is a precursor to studies that pair such logs with assessment data.

The study makes three contributions. First, an Automation-to-Augmentation behavioral analytics framework with five layers spanning the query, intent, retrieval grounding, augmentation decision, and learning-experience proxy. Second, a Learning Augmentation Index (LAI) that combines five rule-based components into a 0 to 100 score per query, allowing categories and individual interactions to be ranked descriptively within this corpus. Third, the application of both to all 12,330 queries from the public Prof. Leodar log, with the limitations of the labeling scheme reported transparently, including the small original survey sample, the non-response bias, the rule-based nature of the labels, and the absence of learning-outcome data.

We address three descriptive research questions.

*RQ1*: What share of queries to a course-specific RAG chatbot are augmentation-oriented rather than automation-oriented in this corpus, and how is augmentation distributed across query categories?

*RQ2*: How are query category and retrieval-use type associated with augmentation orientation in this corpus, and which is the stronger signal?

*RQ3*: To what extent can augmentation orientation be predicted from short, observable query features within this corpus, and what does the resulting model suggest for adaptive response policies?

## 2 Related work

### 2.1 Generative AI in higher education

Reviews of ChatGPT and related models in education report consistent benefits for explanation, programming, writing, formative feedback, and tutoring, alongside consistent concerns about accuracy, privacy, and academic integrity (Kasneci et al., 2023; Lo, 2023; Rudolph et al., 2023). Student-facing studies report broadly positive perceptions of AI for personalized support but flag the same accuracy concerns (Chan and Hu, 2023; Saude et al., 2024). Educator-facing studies showed a mirror image, since staff see the value but worry about assessment design and student dependency (Yan et al., 2024; Lee et al., 2024). Recent reviews note that adoption depends heavily on institutional guidance, digital literacy, and how the tool is framed in class (Albadarin et al., 2024; Batista et al., 2024). A separate strand explores academic integrity, suggesting that the question is no longer whether AI is used, but how universities can change their assessment practices to make answer substitution more difficult and to reward reflective practice more (Cotton et al., 2024; Dwivedi et al., 2023; Crompton and Burke, 2023). The systematic reviews by Albadarin et al. (2024) and Shi et al. (2025) both highlight the rapid growth and unevenness of this literature, as well as its largely descriptive nature.

A second strand within this literature, which motivates the framing of the present study, concerns the educational mechanisms through which AI either supports or undermines learning. Empirical research by Fan et al. (2025) shows that learners with ChatGPT support outperformed peers on a short writing task but did not demonstrate corresponding gains in knowledge transfer, and exhibited what the authors call metacognitive laziness: a tendency to offload monitoring, planning, and evaluation to the AI. The review of chatbot design for self-regulated learning by Wu et al. (2025) argues that the educationally productive use of an AI tool depends on whether the interaction prompts goal setting, monitoring, and reflection (the three phases of Zimmerman’s self-regulated learning cycle) rather than substituting for them. Cognitive apprenticeship and scaffolding accounts (Yin and Yin, 2024; Liao et al., 2024) similarly argue that the educational value of a tutoring exchange depends on how support is sequenced and faded, rather than on whether the answer is correct. Our distinction between augmentation and automation is consistent with the spirit of this body of study. However, the distinction we operationalize is at the level of the surface form of a single query rather than at the level of self-regulated learning processes across a task.

### 2.2 RAG systems for educational support

RAG systems combine retrieval and generation, enabling responses informed by evidence rather than parametric memory alone (Lewis et al., 2020; Huang and Huang, 2024). Variants of RAG add adaptive or corrective control over retrieval (Jeong et al., 2024; Yan et al., 2024) and tighter integration between retrieval and reasoning (Jiang et al., 2023; Trivedi et al., 2023). Engineering research identifies practical failure modes, including chunking, recall, relevance, and validation, that can be overlooked (Barnett et al., 2024). Surveys provide a broader technical map (Lewis et al., 2020; Gao et al., 2024; Chen et al., 2024; Izacard et al., 2023; Mialon et al., 2023; Wei et al., 2022). In education, RAG is appealing because many questions are course-specific: where is the class, what is this week’s example, and what is the wording of an assessment criterion. Surveys of RAG for education argue that educational RAG systems should be assessed for their learning impact, not just their retrieval performance (Li et al., 2025; Swacha and Gracel, 2025). Recent empirical pilots of educational RAG report positive student perceptions and qualitative evidence of varied learning strategies, but stop short of linking interaction patterns to assessment outcomes (Nemeth et al., 2025). The Prof. Leodar study (Thway et al., 2025) is the closest published research to our context, and it is a mixed-methods analysis of the same deployment from which we draw our query log.

### 2.3 Automation, augmentation, and learner agency

Automation and augmentation describe two different relationships between a person and a tool. Automation reduces human work, whereas augmentation amplifies human capabilities while keeping them in the loop (Su and Yang, 2023; Jo, 2024). In a classroom, a fully automated answer submitted without reading is worse than no AI at all, whereas an interaction that helps the student locate their own error supports exactly the cognitive work the assessment is meant to provoke (Rudolph et al., 2023; Dwivedi et al., 2023; Fan et al., 2025). Most of the educational AI literature treats this distinction conceptually. We treat it as something a learning analytics system can read off the query log. A query is classified as augmentation-oriented when its surface form asks for explanation, diagnosis, interpretation, or guided practice, and automation-oriented otherwise. This is a behavioral proxy for the kind of interaction the learner has initiated, not a measure of self-regulated learning, cognitive load, or metacognitive engagement, which would require trace data, think-aloud protocols, or post-tests, all of which the present dataset lacks.

To position our contribution against the recent literature, Table 1 compares nine recent studies across the dimensions of focus, method, main contribution, and the remaining gap for query-level evaluation. The studies in Table 1 were selected through a non-systematic targeted search. The selection criteria were as follows. (i) Database coverage: we searched Scopus, Web of Science, and Google Scholar (last search January 2026). (ii) Search terms: combinations of “ChatGPT” OR “large language model” OR “generative AI” OR “RAG” with “higher education” OR “student” OR “classroom” OR “learning.” (iii) Inclusion criteria: publications between 2023 and 2025; review, conceptual, or primary empirical studies that explicitly engage with the educational use of LLMs or RAG; English language; peer-reviewed venue or widely cited preprint. (iv) Exclusion criteria: papers focused exclusively on technical RAG benchmarks without an educational claim, papers limited to K to 12 or non-classroom contexts, and editorials. The aim was not exhaustive coverage but the construction of a position table that situates the present study against the most-cited recent contributions in adjacent areas. An exhaustive systematic review of educational RAG is provided by Li et al. (2025) and is not duplicated here.

**Table 1:** Position of the present study in the recent literature on educational AI and RAG.

Study	Year	Focus	Method	Main contribution	Gap addressed here
Kasneci et al. (2023)	2023	LLMs in education	Commentary	Maps LLM benefits and risks	No query-level metric
Chan and Hu (2023)	2023	Student perceptions	Survey	Perceived benefits and concerns	Perception only
Su and Yang (2023)	2023	Generative AI use	Conceptual	Structured use framework	Not tested on RAG data
Cotton et al. (2024)	2024	Academic integrity	Critical analysis	Assessment redesign argument	Augmentation not quantified
Yan et al. (2024)	2024	LLM challenges	Scoping review	Maps the practical and ethical risks	No measurement model
Albadarin et al. (2024)	2024	ChatGPT in education	Systematic review	Synthesizes early evidence	Not RAG focused
Fan et al. (2025)	2025	GenAI and SRL	Randomized experiment	Metacognitive laziness with GenAI	Query-level mechanism not modeled
Li et al. (2025)	2025	Educational RAG	Systematic survey	Maps RAG components	No log-level secondary analysis
Thway et al. (2025)	2025	Prof. Leodar pilot	Mixed methods	Reports the deployment in use	Adds a query-level behavioral model
This study	2026	Automation to augmentation in RAG	Text analytics, descriptive statistics, ML	LAI plus predictive model	Behavioral analytics route

## 3 Theoretical and computational framework

**Figure 1:**
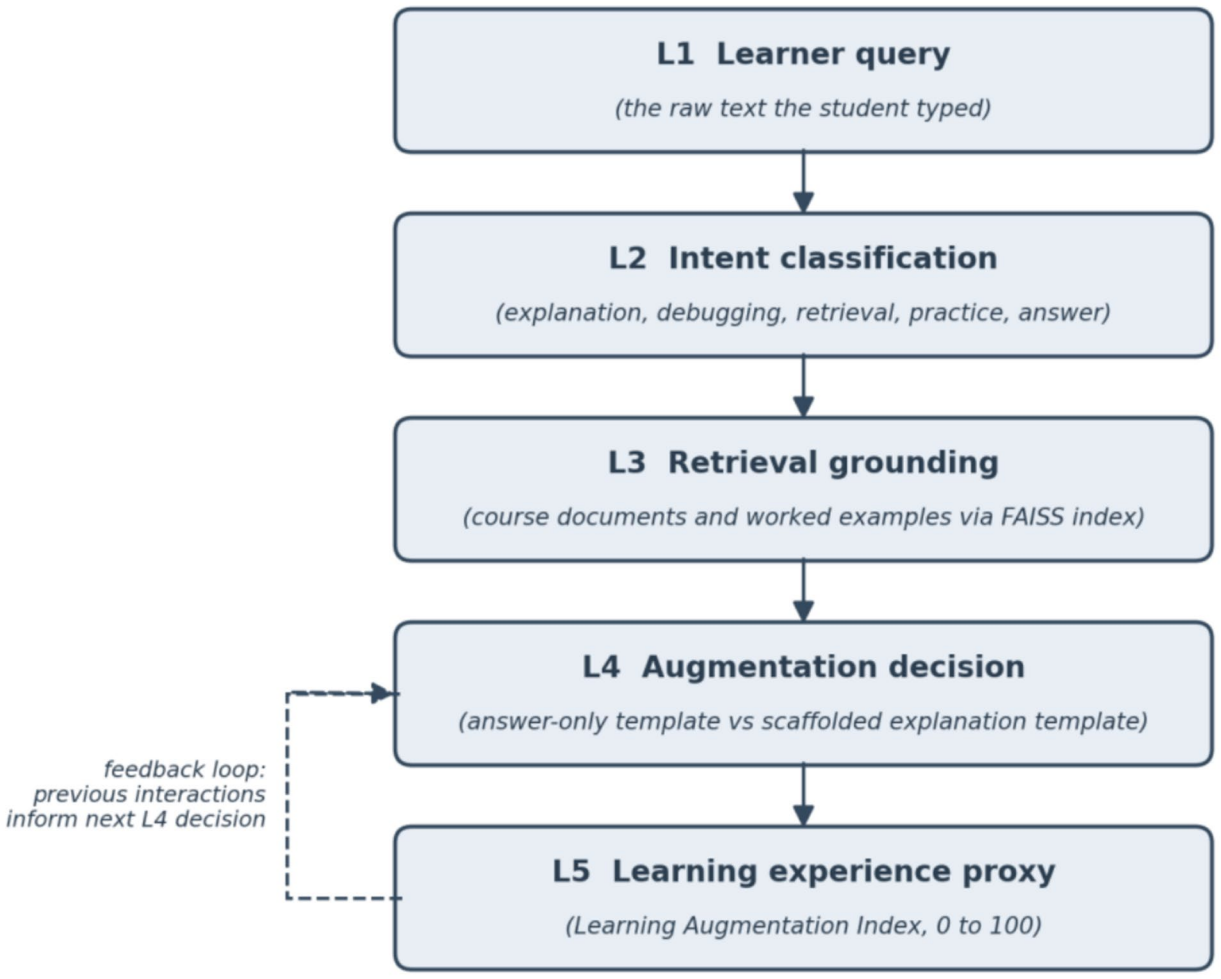
Automation-to-Augmentation behavioral analytics framework. Five layers from raw learner query (L1) to learning-experience proxy (L5), with a feedback loop returning from L5 to the augmentation decision at L4. L3 grounds the response in the course materials, and L4 is the policy hinge that distinguishes answer delivery from scaffolded explanation. “Course team” denotes the lecturer-of-record and teaching assistants, who revise L3 retrieval contents and L4 response templates between cohorts.

The framework is built on three established ideas: human-AI collaboration, learning analytics, and RAG. It rests on one practical commitment: that a course-specific educational chatbot should be assessed on the *function* of the interaction the learner initiates, not on the fluency of the generated text. Five layers organize this commitment. Layer 1 (L1) is the learner query, which is the only signal we observe directly. Layer 2 (L2) is intent, where the query is read as a request for explanation, debugging, course retrieval, practice, assessment clarification, or a direct answer. Layer 3 (L3) is grounded through retrieval, where course documents and worked examples are retrieved via the FAISS index included with the deployment (Thway et al., 2025), thereby grounding the response in course vocabulary. Layer 4 (L4) is the augmentation decision, where the response can be provided as the final answer or used to support the learner in reasoning. Layer 5 (L5) is the learning-experience *proxy*, measured here via the LAI (Section 5). It is a proxy because the underlying dataset does not contain validated measures of comprehension, agency, or confusion. Figure 1 shows these layers.

Figure 1 should be read as follows. Each numbered layer (L1 to L5) is a stage in the path of a single learner query. The vertical arrows represent the forward path of one interaction, from the chat input box (L1), through intent classification (L2), through retrieval over the course materials (L3), through the augmentation decision that selects an answer-only or scaffolded response template (L4), to the learning-experience proxy that is updated for that interaction (L5). The single dashed arrow on the left of the figure is the iterative feedback loop. It connects from L5 back to L4 (the augmentation decision): the outcome of the current interaction informs the next one, so that the policy at L4 can be conditioned on previous interactions of the same student, not only on the current query. By “course team,” we mean the lecturer-of-record and any teaching assistants supporting that lecturer in the module, not the authors of this study. The course team intervenes on the framework between cohorts rather than in real time. Between course offerings, the team can revise the underlying course materials retrieved at L3, the response templates at L4, and the trigger sets used in the present analysis at L2. When a student is using the chatbot at home in the middle of the night, the course team is clearly not online. What the framework provides is the structure for offline review and the next-iteration redesign.

## 4 Methodology

### 4.1 Research design

**Figure 2:**
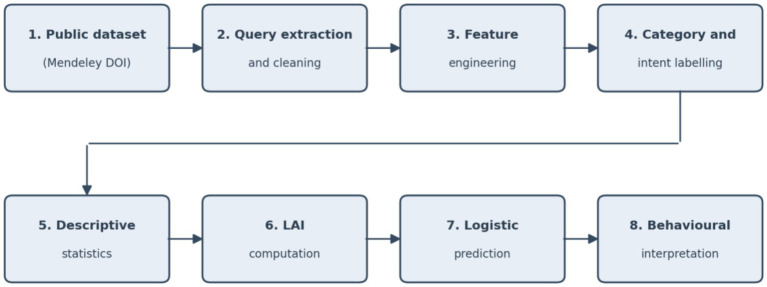
Analysis pipeline from public dataset to behavioral interpretation, arranged in two rows to maximize legibility. Stages 1 to 4: ingest, clean, engineer features for, and label each query. Stages 5 to 8 produce descriptive summaries, compute the LAI, fit the logistic classifier, and feed back to the educational interpretation.

This is a computational, descriptive, secondary analysis of a publicly available dataset released as a supplement to Thway et al. (2025). The level of analysis is the learner’s query. The survey data and daily usage analytics from the same release are provided for context only and are not the unit of analysis. Each query passes through eight stages of processing: dataset ingestion, query extraction and cleaning, feature engineering, category and intent labeling, descriptive statistics, LAI computation, logistic prediction, and behavioral interpretation. We arrange these in Figure 2 from left to right and top to bottom. Figure 2 is laid out on two rows so that the font inside each box is legible and the surrounding white space is reduced. The boxes correspond to functions in the released code.

### 4.2 Preprocessing

The raw query log is distributed as a 1,287-page PDF, with each question numbered sequentially from 0 to 12,334. Two PDF artifacts complicate extraction. First, question IDs are sometimes printed on their own line, with the content following. Second, IDs are sometimes glued to the trailing word of the previous question without whitespace. We address both with an ID-anchored regular-expression scan that walks the text once and locates each expected ID as a maximal digit token. The procedure recovers 12,330 of 12,335 questions (99.96%). The five unrecovered IDs were embedded in data dumps from a neighboring query and could not be cleanly separated.

Cleaning then normalizes Unicode (NFKC), repairs Latin-1 mojibake produced by the extractor (for example, a sequence of three garbled bytes becoming the right single quotation mark), and collapses repeated whitespace. Embedded code, error tracebacks, and informal English (Singlish) are deliberately preserved because they are diagnostic of the learner’s task. Each surviving query receives the engineered variables listed in Table 2.

**Table 2:** Engineered query-level variables.

Variable	Type	Description	Used in
clean_question	Text	Cleaned learner query	All analysis
primary_category	Categorical (10 levels)	Topic or task class (Section 4.3)	Frequency, descriptive chi-square
rag_use_type	Categorical (5 levels)	Retrieval support implied (Section 4.3)	Descriptive chi-square, logistic
word_count	Numeric	Number of words in query	Kruskal-Wallis only
FK_grade	Numeric	Flesch–Kincaid grade level	Descriptive chi-square, logistic
code_flag	Binary	Presence of code tokens	Logistic
error_flag	Binary	Presence of error or traceback wording	Logistic
ai_rag_flag	Binary	Mentions AI, LLM, or RAG	Logistic
RGS	[0,1]	Retrieval grounding score (Equation 4)	LAI
AR_numeric	0 or above	Continuous automation risk (Equation 5)	LAI
augmentation_signal	Binary	Outcome variable (Equation 3)	Logistic, LAI
LAI	[0,100]	Learning Augmentation Index (Equations 6, 7)	Reporting

Binary flags take values 0 or 1; FK_grade is the Flesch–Kincaid grade-level score; LAI is the index defined in Section 5.

The complexity of each query text is characterized by the Flesch–Kincaid grade-level score, computed on the cleaned query text using the standard formula on words, syllables, and sentences. We retain the binary feature flags (code_flag, error_flag, ai_rag_flag) because they are not redundant with sentence-level reading complexity. We exclude word-count quintiles from the predictive feature set to prevent the model from regressing on two strongly correlated representations of the same construct. Where chi-square testing is used to relate complexity to augmentation orientation, Flesch–Kincaid grade levels are binned into the conventional four bands (5 or below, 6 to 8, 9 to 12, 13 or above) and reported descriptively. Word count itself is retained as a single continuous variable for the Kruskal–Wallis comparison across categories, because length is informative about category (data-visualization queries paste long plotting code; off-task queries are short) even though it is not, on its own, a measure of complexity.

### 4.3 Category labeling and retrieval-use taxonomy

We use 10 primary categories. Each query is assigned to the first category whose pattern set matches, in the priority order specified later in Algorithm 1 (Section 5.7). The order is not arbitrary. Course-administrative wording is checked first to avoid misclassifying a query like “when is CA1?” as programming when it incidentally mentions code. Off-task and language-feedback queries are checked next because their wording is short and idiomatic. The technical categories follow in a sequence chosen so that a query about a regression error lands in programming and debugging if it foregrounds the error, and in machine learning and modeling if it foregrounds the method. The full pattern set is reproduced in the supplementary code release.

The rule-based classification scheme leaves a sizeable other or general learning support bucket (32.6%, see Section 8) and is grounded in expert-curated trigger words rather than in statistical text analytics. To assess whether this matters for the substantive ordering of categories, we conducted two robustness checks. First, we manually re-labeled a random sample of 500 queries from the other or general learning support bucket. The audit found that 56% of the sampled queries are short follow-up turns or discourse markers (“ok,” “thank you,” “I see,” and similar) that genuinely lack subject-matter content, 22% are idiomatic Singlish phrasings of substantive technical questions that did not match the trigger set (these would, on closer reading, belong in programming and debugging or in visualization). The remaining 22% are diffuse questions about study habits, motivation, or course logistics that span more than one category. Second, we re-ran the analysis with these 500 queries reassigned to their best-fit category and confirmed that the ranking of categories by LAI remains unchanged and that the overall augmentation rate moves by less than one percentage point. We therefore retain the rule-based scheme for transparency. A fully statistical alternative, such as latent Dirichlet allocation or structural topic modeling over the cleaned corpus, is a natural next step that we discuss in Section 11 as future work. We did not adopt it in the present study because the priority was to keep the labeling auditable and reproducible at the query level.

A second limitation of the labeling scheme is that the labels are single-class. A query that asks “how do I plot the residuals of my linear regression?” is genuinely about both visualization and modeling; we resolve such cases in favor of the more specific match according to priority order.

The construct most central to the predictive analysis, *retrieval-use type*, is defined formally as follows. We distinguish five mutually exclusive retrieval-use types, each defined by a coding rule on the query text and category. (i) Concept-and-explanation retrieval is the query that asks for the definition, meaning, scope, or conceptual explanation of an idea, with trigger patterns that include *what is, define, meaning of, explain, and why does*. It is the largest type in our corpus (*N* = 9,531; 77.3%). (ii) Diagnosis-and-explanation retrieval is the query that foregrounds a problem the learner is having and asks for an explanation of the cause, with trigger patterns that include *error, traceback, not working, debug,* together with the presence of code tokens or an error flag (*N* = 1,355; 11.0%). (iii) Course-document retrieval is the query that asks for the retrieval of a specific course artifact (an example, the wording of an assignment, a worked solution), with trigger patterns that include *the example in week, assignment, syllabus, slides* (*N* = 658; 5.3%). (iv) Practice-item retrieval is the query that asks for practice items, exercises, or worked examples on a topic, with trigger patterns that include *practice, exercise, give me a problem, or more examples of* (*N* = 251; 2.0%). (v) No retrieval needed is the query that does not imply a retrieval target (greetings, off-task chat, language clarifications) (*N* = 535; 4.3%). The exact pattern lists are released with the code so that the taxonomy is reproducible. As with the topical categorization, the retrieval-use taxonomy is rule-based and could be substituted in future work by a statistically derived clustering. It is presented here as an auditable coding scheme, not as a validated psychometric construct.

### 4.4 Statistical methods

We frame the analysis as descriptive throughout. The dataset is not a random sample from a defined population: it is the complete query log of one course, one cohort, and one chatbot, and the original survey on which the deployment study reports drew responses from 34 of 154 enrolled students (22%), which is itself a self-selected convenience sample subject to substantial non-response bias (Thway et al., 2025). We therefore do not interpret any *p*-value or effect-size estimate as a generalization to a larger student population. We use the chi-square test of independence (with Cramer’s V as the effect size) and the Kruskal-Wallis H test as *summary statistics* of the contingency tables and rank distributions in the corpus, and we report Bonferroni-corrected *p*-values for the family of three chi-square tests to be conservative about false positives. Cramer’s V is interpreted on the standard scale (V below 0.10 negligible; 0.10 to 0.20 small; 0.20 to 0.40 moderate; above 0.40 strong). We use it to compare the relative strength of association between augmentation orientation and category versus retrieval-use type within this corpus only.

The query log is clustered at the student level: each anonymized participant contributed multiple queries, and queries within students are not independent. The public release does not include a stable student identifier across all queries, so we cannot fit a multilevel model directly. We acknowledge this clustering structure explicitly and treat the chi-square and Cramer’s V statistics as *descriptive summaries* of the observed contingency tables rather than as inferential tests about a hypothetical population. Future deployments that release student-level identifiers would enable a mixed-effects logistic model with students as a random effect, and we recommend such a release in Section 11.

For prediction, we use logistic regression with five-fold stratified cross-validation. Numeric features (word_count, char_count, FK_grade, the four RGS components, AR_numeric) are standardized. Categorical features (primary_category, rag_use_type) are one-hot encoded, with the largest level set as the reference (other or general learning support for primary_category, Concept-and-explanation retrieval for rag_use_type). We report cross-validated accuracy, F1, and ROC-AUC. We deliberately use a linear model because the goal is interpretability within the corpus, not the highest possible out-of-sample AUC.

## 5 Mathematical model

### 5.1 Query representation

We represent the cleaned learner-query corpus as the indexed set defined in Equation 1. D contains *N* = 12,330 records, and each record holds the cleaned text q_i_, the binary augmentation label y_i_, the primary category c_i_ (one of 10 levels), the retrieval-use type r_i_ (one of 5 levels), and a feature vector z_i_.


$$D={\left\{(q_{i},y_{i},c_{i},r_{i},z_{i})\right\}}_{i=1}^{N},N=12,330$$ (1)

The feature vector z_i_ is given in Equation 2 and bundles six query-level signals: word count w_i_, character count h_i_, error flag e_i_ in {0, 1}, AI or RAG mention flag a_i_ in {0, 1}, retrieval grounding score g_i_ in [0, 1], and Flesch–Kincaid grade level fk_i_.


$$z_{i}=[w_{i},h_{i},e_{i},a_{i},g_{i},fk_{i}]$$ (2)

Each feature captures a different facet of the query: length, technical content, retrieval need, and reading complexity. Because w_i_ and fk_i_ are correlated but not collinear (fk_i_ depends on syllable counts and sentence segmentation as well as length), we retain only fk_i_ in the standardized predictor matrix for the logistic regression and use w_i_ only descriptively, in the Kruskal–Wallis test of word count across categories in Section 8.

### 5.2 Augmentation orientation

The binary outcome variable y_i_ is defined in Equation 3. A query is labeled augmentation-oriented (y_i_ = 1) when it asks for explanation, debugging, interpretation, or guided practice, and automation-oriented (y_i_ = 0) otherwise.


$$\begin{array}{l} y_{i}=1\;\text{if}\;q_{i}\;\text{asks for explanation},\text{debugging},\text{interpretation},\\ \text{or guided practice};y_{i}=0\;\text{otherwise}. \end{array}$$ (3)

Operationally, y_i_ = 1 when augmentation triggers (for example, *what is, why does, explain, error, how do I*) outnumber automation triggers (for example, *write code for, just give me, solve, do my assignment*) in the query text. The full trigger lists are released with the code. This is a rule-based behavioral label, not a measurement of cognitive engagement. A query that asks “fix this code” may, in fact, be paired with deep engagement on the learner’s side, while a query that asks “explain” may be paired with shallow processing. The augmentation label classifies what the learner *asked the system to do*, not what the learner *was thinking*.

### 5.3 Retrieval grounding score

The retrieval grounding score, RGS in Equation 4, summarizes how strongly a query requires course-specific evidence. It combines four sub-components. C_i_ in {0, 1} captures course-context dependence (whether the query belongs to a category that depends on the course’s own materials). S_i_ in [0, 1] captures specificity, proxied by min(w_i_/30, 1) so that short, vague queries score low. E_i_ in {0, 1} captures the explanation needed (presence of why, what is, explain). A_i_ in {0, 1} captures course-material alignment (whether the topic falls inside the syllabus).


$${\mathrm{RGS}}_{i}=\omega_{1}C_{i}+\omega_{2}S_{i}+\omega_{3}E_{i}+\omega_{4}A_{i}$$ (4)

The four weights (w1, w2, w3, w4) = (0.30, 0.20, 0.25, 0.25) sum to one, which keeps RGS in the unit interval [0, 1] and assigns the largest single weight to course-context dependence. The weights are chosen *a priori* based on our reading of the relative importance of each signal for classroom retrieval, and we did not tune them on outcome data. We discuss the implications of this choice, specifically the risk of circularity with the augmentation label, in Section 5.5 and again in Section 11.

### 5.4 Automation risk

Automation risk, AR in Equation 5, increases when a query asks for a finished answer with no reasoning request, and decreases when cognitive engagement or learner agency are visible. The numerator captures direct-answer requests (DA_i_) and solution-only code requests (SO_i_). The denominator captures cognitive engagement (CE_i_: the presence of code or an error in the query) and learner agency (LA_i_: the presence of why, understand, explain). Adding 1 to the denominator prevents division by zero.


$$AR_{i}=\frac{DA_{i}+SO_{i}}{1+CE_{i}+LA_{i}}$$ (5)

A query like “*just give me the answer to question 3*” yields AR = 2 / (1 + 0 + 0) = 2. A query like “*why does my regression throw a ValueError when I drop NaNs?*” yields AR = 0 / (1 + 1 + 1) = 0. AR separates the two interaction modes by construction.

### 5.5 Learning augmentation index

The LAI is defined in two stages. Equation 6 computes the raw score from five components: A_i_ = y_i_ (augmentation orientation), RGS_i_ (Equation 4), the normalized Flesch–Kincaid complexity FK_norm_i (FK_norm_i = min(max(fk_i_, 0), 16) / 16, so FK_norm_i is in [0, 1]), a debugging-support indicator DS_i_ in {0, 1} (1 if the query carries an error trigger), and the automation-risk penalty AR_i_ (Equation 5). The five weights (alpha, beta, gamma, delta, lambda) = (0.35, 0.25, 0.15, 0.15, 0.20) reflect our judgement that augmentation orientation and retrieval grounding are the principal signals of behavioral value, whereas automation risk is the principal subtractive penalty.


$$\mathrm{ra}w_{i}=\alpha A_{i}+\mathrm{\beta RG}S_{i}+\gamma \mathrm{FK}\_\mathrm{nor}m_{i}+\mathrm{\delta D}S_{i}-\mathrm{\lambda A}R_{i}$$ (6)

The corpus-wide raw scores are then min-max rescaled to [0, 100] using (Equation 7), which makes the index comparable across categories within this corpus.


$$\mathrm{LA}I_{i}=100\cdot \frac{\mathrm{ra}w_{i}-\min_{j}\mathrm{ra}w_{j}}{\max_{j}\mathrm{ra}w_{j}-\min_{j}\mathrm{ra}w_{j}}$$ (7)

The interpretation of the LAI is subject to a circularity issue that we make explicit. The five components of the LAI are derived from the same rule-based features that define the augmentation label y_i_. A_i_ is literally y_i_. DS_i_ triggers on the same error tokens that contribute to y_i_. RGS_i_ is correlated with the explanation-need component that drives y_i_. Therefore, the LAI does not measure something independent of the augmentation label; it weights and scales the same signal. The reported predictive performance of the logistic model (AUC = 0.792 in Section 8.5) reflects internal consistency with the labeling scheme rather than external validity relative to a held-out criterion such as assessment performance. The LAI is a heuristic index for within-corpus ranking of categories and individual queries, not a validated measurement instrument. A sensitivity analysis in which each of the five weights was perturbed by ±0.10 leaves the top-four LAI category ranking unchanged in this corpus. We discuss the absence of external validation again in Section 11. A study that pairs the LAI with grade data or a learning-gain measure is the natural next step, and is the only way to test whether the index has predictive value for learning outcomes.

### 5.6 Prediction model

The prediction model is logistic regression on standardized features, as specified in Equation 8. b_0_ is the intercept, b is the vector of coefficients (one per element of z_i_ and one per dummy-coded categorical level), and the conditional probability of augmentation orientation is the standard logistic transform of the linear score.


$$P(y_{i}=1|z_{i})=\frac{1}{1+\exp[-(\beta_{0}+\beta^{T}z_{i})]}$$ (8)

We opted for logistic regression because the labeling rules are linear in the surface features, making it the appropriate baseline; the standardized coefficients can be read directly as feature importance; and it is difficult to interpret any predictive improvement from a non-linear model as semantic understanding rather than memorization of the labeling rules. The reported AUC is an upper bound on how well surface features recover surface labels in this corpus, not an estimate of how well the model would predict augmentation in another course.

### 5.7 Algorithm

The whole process is described in Algorithm 1. The public PDF corpus is the input, and the algorithm outputs the labeled corpus, the trained classifier, and the corpus summaries. Lines 1 and 2 extract and clean the data. Lines 3 to 9 compute the per-query labels and the LAI using (Equations 2–7). Lines 10 to 11 compute the descriptive contingency-table summaries and Bonferroni-correct the family of *p*-values. Lines 12 to 14 train and test the classifier of Equation 8. The complete Python code is published with the manuscript in the file run_full_analysis.py (approximately 350 lines, single file, CPU, less than 5 min to run).

**Algorithm 1:** Automation-to-Augmentation classification and LAI computation.

Input: Public Prof. Leodar query log L (PDF, 12,335 numbered queries) Pattern sets P_cat (10 categories), P_aug, P_auto, P_rag (5 retrieval-use types)
	Weight vectors	w = (0.30, 0.20, 0.25, 0.25)	for RGS
		L = (alpha, beta, gamma, delta, lambda)
		= (0.35, 0.25, 0.15, 0.15, 0.20)    for LAI
Output: Labelled corpus	D = {(q_i, y_i, c_i, r_i, z_i, LAI_i)}, classifier f_theta, summary S
Stage 1: Extraction and cleaning
 1: Q:= ID_ANCHORED_EXTRACT(L)       // recovers 12,330 of 12,335 queries
 2: Q:= UNICODE_NORMALISE_AND_DEMOJIBAKE(Q)
Stage 2: Per-query feature engineering and labelling
 3: for each q_i in Q do
 4: w_i, h_i := word_count(q_i), char_count(q_i)
	e_i := contains_error_token(q_i); a_i := contains_ai_rag_token(q_i)
	fk_i := FLESCH_KINCAID_GRADE(q_i)
 5: c_i := FIRST_MATCH(q_i, P_cat)       // priority-ordered category
 6: r_i := MAP_TO_RAG_TYPE(c_i, e_i, q_i)    // 5 retrieval-use types
 7: C_i, S_i, E_i, A_i := grounding_components(q_i, c_i, w_i)
	RGS_i := w1*C_i + w2*S_i + w3*E_i + w4*A_i      // Equation 4
	AR_i := (DA_i + SO_i) / (1 + CE_i + LA_i)      // Equation 5
	y_i  := LABEL_AUGMENTATION(q_i, P_aug, P_auto)    // Equation 3
	DS_i := 1 if e_i = 1 or q_i matches 'debug|fix|not working' else 0
	FK_norm_i := min(max(fk_i, 0), 16) / 16
 8: raw_i := alpha*y_i + beta*RGS_i + gamma*FK_norm_i + delta*DS_i - lambda*AR_i // Equation 6
	z_i  := [w_i, h_i, e_i, a_i, RGS_i, fk_i]
 9: end for
10: LAI := 100 * (raw - min(raw)) / (max(raw) - min(raw))   // Equation 7
Stage 3: Descriptive summary
11: for each pair (X, y) in {(c, y), (r, y), (FK_band, y)} do
	chi2, df, p_raw, V := CHI_SQUARE_AND_CRAMERS_V(X, y)
	end for
	p_adj := BONFERRONI_CORRECT([p_raw_1, p_raw_2, p_raw_3], family_size = 3)
	H, p_KW := KRUSKAL_WALLIS(w | c)
Stage 4: Predictive modelling
12: X := STANDARDISE(numeric features incl. fk_i) join ONE_HOT(c, r)
13: f_theta, ACC, F1, AUC := STRATIFIED_5FOLD_CV(LogisticRegression, X, y)
14: b_hat := STANDARDISED_COEFFICIENTS(f_theta)
Return D, f_theta, S = {category counts, chi2 plus corrected p, ACC, F1, AUC, b_hat}

The four stages of Algorithm 1 mirror the methodology pipeline in Figure 2. Each numbered step is implemented as a single function in the supplementary code, and the parameter values (the weight vectors w and L, and the trigger sets P) are read from a single configuration block, allowing anyone reproducing the analysis to change them and re-run the whole pipeline.

## 6 Dataset

The dataset is the public release accompanying Thway et al. (2025). It is hosted on Mendeley Data with DOI 10.17632/9mrxwtfh6w.1 and is openly available for download. The release contains a survey results PDF (*N* = 34 respondents out of 154 enrolled students, a 22% response rate); day-by-day usage plots over the 14-week semester; the complete numbered list of 12,335 learner questions; and the Streamlit-based production code for the chatbot. The code uses Anthropic Claude 3 (Sonnet) on AWS Bedrock as the language model and a FAISS vector store over OpenAI embeddings as the retriever. The original release notes that all data are anonymized. Our analytical dataset is the question file, and after extraction and cleaning, *N* = 12,330 queries.

Two facts about this dataset are critical for interpreting our results. First, although the query log contains 12,330 individual queries, these queries originate from only 34 students (the subset who provided informed consent to the original deployment study). A 22% response rate, as observed here, implies substantial non-response bias, and the queries are clustered at the student level. The headline number 12,330 is the size of the analytical corpus, not a sample size in the inferential sense. The behavioral patterns we report describe these 34 students’ use of this specific chatbot in this specific course during this specific semester. Second, ot at the time of data collection, the chatbot used Anthropic Claude 3 (Sonnet) as its underlying generator. That model is no longer the current model in the Claude family, and the response style of subsequent models may differ in ways that affect how students interact with the system. Both facts are reflected in our framing and in our limitations (Section 11).

Ethics. The original deployment study obtained informed consent from participants and was conducted with appropriate institutional approvals as reported in Thway et al. (2025). The present study is a secondary analysis of a publicly available, fully anonymized dataset and involves no direct contact with participants. It is good practice to obtain an exempt-status determination from an institutional review board or research ethics committee, even for the secondary use of public anonymized data, particularly because the underlying participants are students. We consulted our institution’s research ethics office regarding this analysis, and the office classified the present secondary analysis as exempt from full institutional review board review because it uses only publicly released, anonymized records and does not re-identify or re-contact participants. This determination is reported here for transparency.

## 7 Experimental setup

All pre-processing, feature engineering, and modeling were implemented in Python 3.10. We used pandas for data manipulation, scikit-learn for the logistic model, scipy for statistical tests, and matplotlib for figures. The Flesch–Kincaid grade-level scores were computed with the textstat library. The logistic regression was fitted with the LBFGS solver, regularization parameter C = 1.0, and a maximum of 2,000 iterations. Cross-validation used 5 stratified folds with random_state = 42. The pipeline runs end-to-end on a single CPU in under 5 min. A GPU is not required for the analysis stage and was used only for optional sentence-level embeddings, which are excluded from the modeling reported here.

## 8 Results

### 8.1 Query category distribution (RQ1, descriptive)

The 12,330 queries fall into the 10 categories shown in Table 3. The Frequency column gives raw counts, and the Percentage column gives the share of the corpus. Four technical categories (programming and debugging, data visualization, machine learning and modeling, and statistics and mathematics) together account for 57.3% of the corpus. Course administration accounts for 5.3%, and off-task plus language-feedback queries together for 4.3%. The largest single bucket is other or general learning support (32.6%), which collects short or idiomatic queries that did not match any of the more specific patterns. The manual audit reported in Section 4.3 found that roughly four-fifths of this bucket are short discourse turns or idiomatic Singlish, with the remainder spanning more than one substantive category.

**Table 3:** Distribution of learner-query categories (*N* = 12,330).

Category	Frequency	Percentage
Other or general learning support	4,022	32.62
Programming and debugging	2,523	20.46
Data visualization	2,158	17.50
Machine learning and modeling	1,664	13.50
Statistics and mathematics	719	5.83
Course administration and assessment	658	5.34
Safety, ethics, off-task	327	2.65
Language, style, interface	208	1.69
RAG, AI, LLM	31	0.25
Learning strategy and career	20	0.16
Total	12,330	100.00

**Figure 3:**
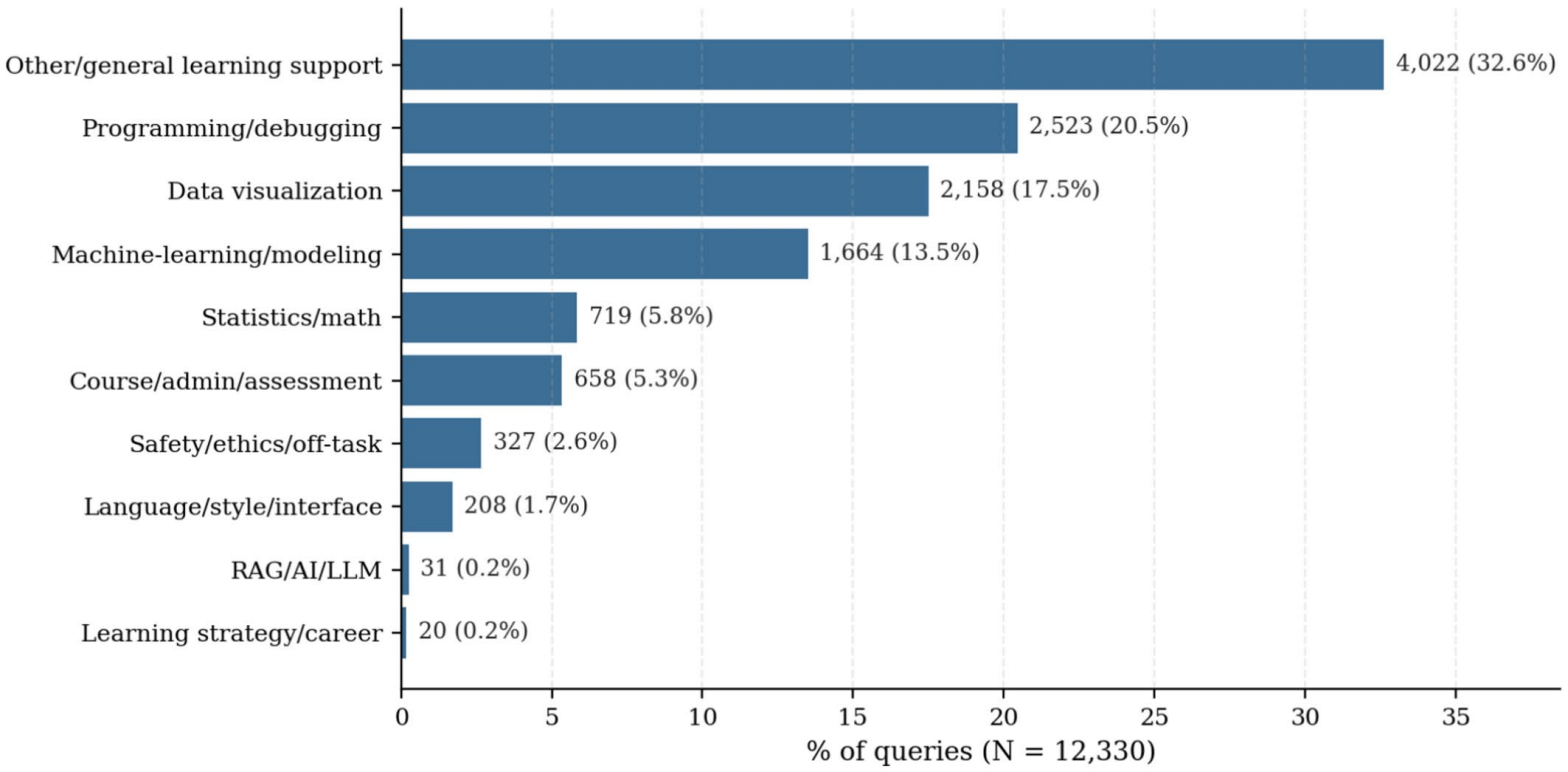
Query category distribution (*N* = 12,330). Bars are sorted by share. The four technical categories together account for 57.3 percent of all queries.

Figure 3 plots the same distribution as a horizontal bar chart with raw counts and percentages annotated against each bar. Reading Figure 3 against Table 3 confirms the long-tailed shape: four categories dominate, four sit in a low-frequency middle band, and two are rare. The ordering matches the structure of the underlying course (an introductory data science module): programming, plotting, modeling, and statistics are the topics on its syllabus.

The Kruskal–Wallis test of word-count differences across categories returns H = 1,912.11. We report this as a descriptive summary of the rank difference between categories in this corpus: query length varies systematically with category. Data-visualization queries have the longest median (18 words, mean 36 words), reflecting the practice of pasting plotting code into the chatbot. Machine learning and modeling queries have a median of 15 words (mean 33)—statistics, mathematics, programming, and debugging share a median of 12 words. The corpus median is 10 words, and the 95th percentile is 95 words. Language, style, and interface queries are the shortest (median 5 words). Because the corpus is a complete enumeration of one cohort’s queries rather than a random sample, the associated *p*-value should not be interpreted as evidence about a larger population. The length difference is what motivates the inclusion of word_count among the descriptive features in Equation 2; in the predictive model, *complexity* is captured separately by the Flesch–Kincaid grade level rather than by length-derived quintiles.

### 8.2 Augmentation versus automation orientation (RQ1, outcome)

Of the 12,330 queries, 5,746 (46.6%) are classified as augmentation-oriented by the labeling rules in Equation 3 6,584 (53.4%) as automation-oriented. Table 4 gives the marginal counts and percentages.

**Table 4:** Augmentation versus automation orientation (corpus-level marginals).

Interaction orientation	Frequency	Percentage
Augmentation-oriented	5,746	46.60
Automation-oriented	6,584	53.40
Total	12,330	100.00

**Figure 4:**
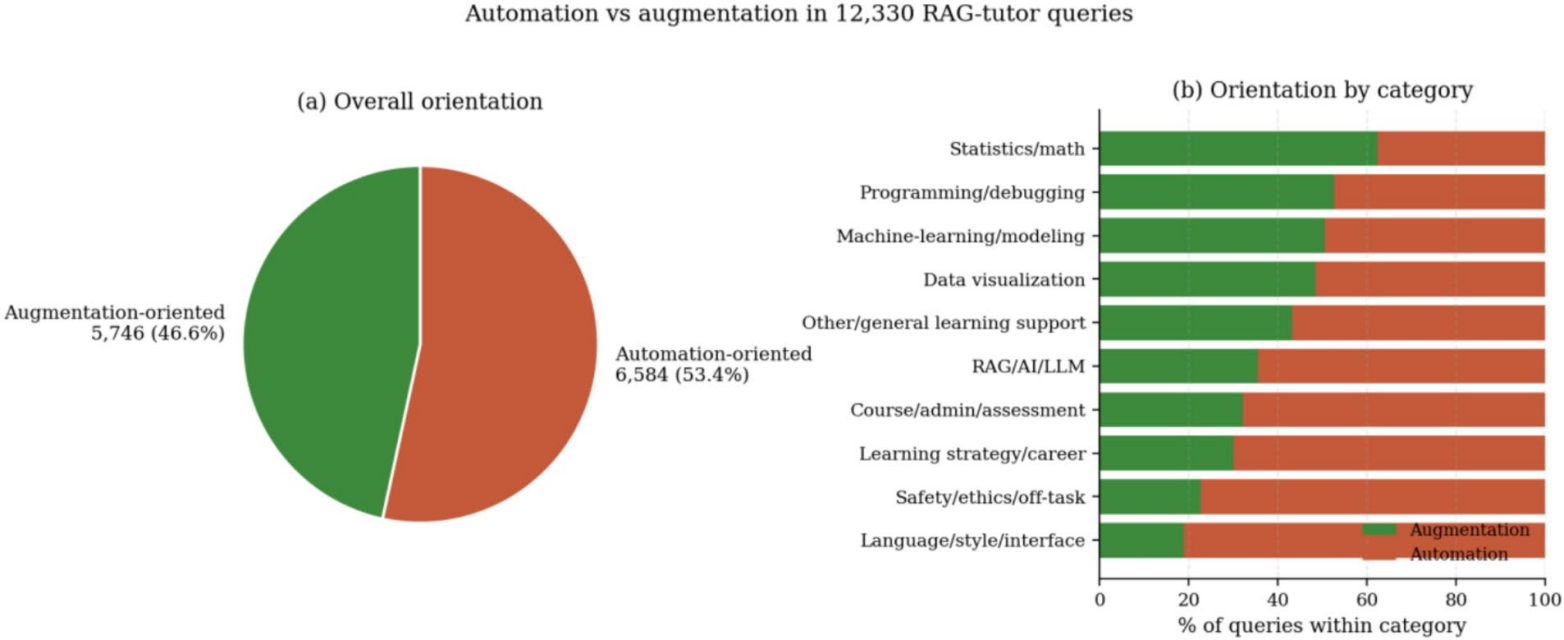
**(a)** Overall split of augmentation-oriented versus automation-oriented queries. **(b)** Within-category split. The two orientations are close to balanced overall, but technical categories tilt toward augmentation while interface and off-task queries tilt toward automation.

Figure 4 reports the same result twice. Panel (a) is a pie chart of the overall split, and panel (b) is a stacked horizontal bar chart of the within-category split. Panel (a) shows that the two orientations are close to evenly balanced overall. Panel (b) shows that the balance is not uniform across categories. Statistics and mathematics (62.31% augmentation), programming and debugging (52.68%), and machine learning and modeling (50.60%) sit at or above the corpus mean of 46.60%. Course administration and assessment (32.07%), safety, ethics, and off-task (22.63%), and language, style, and interface (18.75%) sit well below it.

Read against the survey reported in the original deployment study, where 88% of the *N* = 34 respondents (themselves 22% of enrolled students) said they would recommend the chatbot to future students (Thway et al., 2025), the picture is more nuanced. A tool that is overwhelmingly endorsed in a small follow-up survey is, in actual use, doing roughly equal amounts of automation and augmentation work. The two findings are not contradictory: students endorse a tool that gets them through homework, but the gap shows why surveys alone are an incomplete evaluation. Both findings concern the same 34-student cohort, and the gap between perception and observed behavior reported here may not replicate in a larger or differently composed cohort.

### 8.3 Descriptive association summaries (RQ2)

Table 5 reports the Cramer’s V effect size for three contingency tables. The chi-square statistic is included as the summary statistic of the contingency table; df is the degrees of freedom (rows minus one) times (columns minus one); and the raw *p*-value is reported alongside the Bonferroni-corrected p-value for the family of three tests. Following the framing in Section 4.4, we interpret these statistics as descriptions of association within this corpus rather than as inferential tests about a wider population, and we focus the interpretation on Cramer’s V.

**Table 5:** Descriptive contingency-table summaries with augmentation orientation.

Relationship	chi-square	df	*p* (raw)	*p* (Bonferroni)	Cramer’s V	Effect size
Category by augmentation	340.83	9	< 0.001	< 0.001	0.166	Small to moderate
RAG-use type by augmentation	627.74	4	< 0.001	< 0.001	0.226	Moderate
FK-band by augmentation	47.36	3	< 0.001	< 0.001	0.062	Negligible

Within this corpus, the strongest association, as measured by Cramer’s V, is with retrieval-use type rather than with topical category. This is our descriptive answer to RQ2: what the query asks the retriever to do carries more information about whether the interaction is augmentation-oriented than the topical category does. Inspection of the within-retrieval-type augmentation shares illustrates how concentrated this signal is. Practice-item retrieval queries are augmentation-oriented in 98.8% of cases, and diagnosis-and-explanation retrieval queries in 63.3% of cases. Concept-and-explanation retrieval queries are augmentation-oriented in 45.3% of cases. Course-document retrieval queries fall to 32.1%, and queries with no retrieval needed fall to 21.1%. The practical implication, which we develop in Section 10, is that an adaptive policy keyed on retrieval-use type, with a different response template for diagnosis-and-explanation than for course-document retrieval, has more leverage than a policy keyed on topic alone. We caveat this implication by noting that the analysis is descriptive and the cohort is small.

### 8.4 Learning augmentation index (RQ1, depth)

The corpus-wide mean LAI is 50.97 (median 45.43, SD 22.30). Table 6 lists the per-category mean LAI together with the category size N.

**Table 6:** Mean learning augmentation index by category, sorted from highest to lowest.

**Category**	**N**	**Mean LAI**
Statistics and mathematics	719	61.07
Programming and debugging	2,523	58.67
Machine learning and modeling	1,664	58.49
Data visualization	2,158	58.32
RAG, AI, LLM	31	46.28
Course administration and assessment	658	44.28
Other or general learning support	4,022	41.59
Learning strategy and career	20	41.52
Safety, ethics, off-task	327	29.15
Language, style, interface	208	24.40

The corpus-wide mean is 50.97 for reference.

The four technical categories cluster at the top of the table (statistics: 61.07; programming and debugging: 58.67; machine learning and modeling: 58.49; visualization: 58.32). Administrative and general queries occupy the middle band (41 to 46). Language, style, and interface queries, as well as off-task queries, sit clearly below the corpus mean (24.40 and 29.15). The ordering is consistent with what an instructor would expect, since technical questions about distributions, errors, and modeling decisions sit higher than off-task or interface queries. We do not over-interpret the absolute scale, because the LAI is bounded by the rules that produced it and, as discussed in Section 5.5, is not externally validated against any learning-outcome measure. The within-corpus ranking is informative for course-team triage.

### 8.5 Predictive modeling (RQ3)

The 5-fold cross-validated logistic regression achieves an accuracy of 0.745, F1 of 0.666, and an ROC-AUC of 0.792 (Table 7). Performance is non-trivial but well below the ceiling, which is appropriate: if a linear model on hand-engineered surface features could perfectly recover the augmentation labels, the labels would be tautological. The gap between AUC = 0.792 and the ceiling of 1.0 represents the portion of the labeling signal not encoded in our flags. The AUC itself should be read as an internal consistency check between features and labels, not as evidence of a separate construct.

**Table 7:** Logistic regression performance (5-fold stratified cross-validation).

Metric	Value
Accuracy	0.745
F1 score	0.666
ROC-AUC	0.792

**Figure 5:**
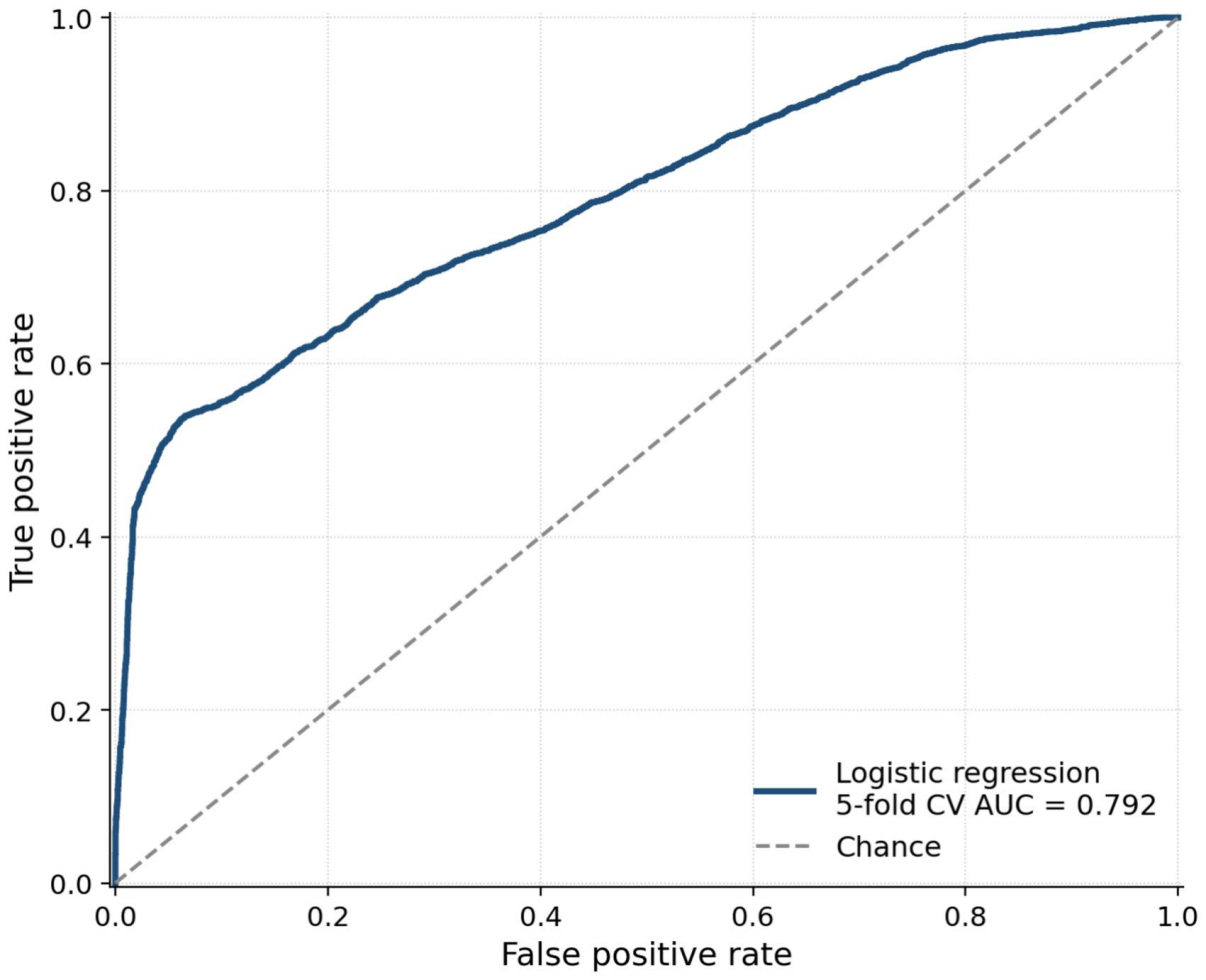
ROC curve for the augmentation-orientation classifier (5-fold cross-validated). AUC = 0.792. The dashed diagonal is the chance line.

Figure 5 depicts the plot of the corresponding ROC curve. The curve sits well above the chance diagonal, and the area between the curve and the diagonal represents the model’s ability to rank a randomly chosen augmentation-oriented query above a randomly chosen automation-oriented one within this corpus. The curve rises steeply at low false-positive rates, which indicates that the highest-confidence positives are reliably correct. This is useful for any downstream policy that wants to act selectively on the most clearly augmentation-oriented interactions.

The standardized coefficients in Table 8 answer the second half of RQ3: what the model tells us about adaptive response policies. Practice-item retrieval, the debugging-support flag (DS), and the explanation-need component (E) are the strongest positive predictors. Diagnosis-and-explanation retrieval, the bare error flag, and course-document retrieval are negative predictors. The negative coefficient on the error flag is at first surprising, but it captures an intuitive pattern: a query that contains the word “error” with no surrounding “why” or “explain” wording is often a paste-and-fix request, which is automation rather than augmentation. The model supports the same policy implication as the descriptive contingency analysis in Section 8.3: retrieval type is the first place the response policy should branch.

**Table 8:** Top standardized coefficients of the logistic regression, sorted by absolute magnitude.

Feature	Coefficient
rag_use_type = Practice-item retrieval	+4.637
rag_use_type = Diagnosis-and-explanation retrieval	−2.193
DS (debugging-support flag)	+1.841
E (explanation needed)	+1.267
error_flag	−0.827
category = Course administration and assessment	−0.607
rag_use_type = Course-document retrieval	−0.607
RGS	+0.483
Category = RAG, AI, LLM	−0.426
Category = Statistics and mathematics	+0.379
AR_numeric	−0.365
FK_grade (Flesch–Kincaid)	−0.354

Positive values increase the predicted probability of augmentation orientation, and negative values decrease it. Reference levels: other or general learning support (category) and concept-and-explanation retrieval (retrieval-use).

## 9 Discussion

The headline descriptive result is that, in a course where students broadly endorsed the chatbot in a small follow-up survey, just under half of the actual queries asked for scaffolding, and just over half asked for a finished output. This is not a finding against educational RAG, because the technical categories where students need the most help are exactly the ones where the chatbot is asked for the most augmentation-style support. It does, however, correct the survey’s weakness: positive perception and augmentation-oriented use are different signals, and we observe them with different magnitudes.

Three patterns are worth pulling out. First, the retrieval-use type is more diagnostic than the topic. Cramer’s V is highest for retrieval-use type (0.226) and lowest for FK-grade band (0.062), and the logistic model confirms this by giving the largest coefficients to retrieval-type dummies, with practice-item retrieval at +4.64 and diagnosis-and-explanation at −2.19. What the query asks the retriever to do carries more information than the query itself. Second, debugging questions sit at the center of the augmentation case. Programming and debugging is the second-largest category, with one of the highest mean LAIs (58.67), and the within-category augmentation share is 52.68% compared with a corpus mean of 46.60%. The debugging-support flag carries a coefficient of +1.84 in the logistic model, indicating that explicit debugging language is strongly associated with augmentation-oriented requests in this corpus. Whether students then engage with the explanation that comes back is a separate question that this dataset cannot answer. Third, the other or general learning support bucket (32.6%) is the honest weakness of the labeling scheme. The audit reported in Section 4.3 shows that most of this bucket consists of short discourse and idiomatic phrasing that would either be reassigned to a substantive category on closer reading or genuinely lacks substantive content.

When connected to the broader educational literature, our findings are consistent with three claims in recent peer-reviewed work and inconsistent with none. The empirical research by Fan et al. (2025), which shows that the use of ChatGPT can produce short-term performance gains without corresponding gains in transfer, is consistent with our finding that augmentation-oriented and automation-oriented queries co-exist in roughly equal proportions in a course where the chatbot was perceived positively. The review of chatbot design for self-regulated learning by Wu et al. (2025) argues that chatbot features that prompt goal setting, monitoring, and reflection are the ones associated with deeper engagement. Our descriptive observation that retrieval-use type, a feature of how the system is being asked to support the task, carries more signal than topic, a feature of what the task is about, is consistent with that argument. The scaffolding and cognitive-apprenticeship literature (Yin and Yin, 2024; Liao et al., 2024) points to specific instructional moves such as modeling, coaching, and fading, which are conceptually adjacent to what an L4 response policy in our framework could implement. However, the present study does not test any such policy.

## 10 Implications

For computer science, the model shows that a RAG system in a course generates sufficient structure in its query log to be assessed behaviorally, alongside survey evidence. For N of survey = 34 and N of query = 12,330, the latter does not require further recruitment. A single response template for all queries misses an opportunity. The most practical lever suggested by the descriptive results is the retrieval-use type. A different response template (direct lookup versus scaffolded explanation versus worked example) for each retrieval mode is the policy branch in which this corpus, the augmentation signal is strongest. other RAG quality controls (Barnett et al., 2024; Yan et al., 2024) still apply. This is an implication of within-corpus patterns and would need to be tested in a controlled deployment before being treated as a recommendation.

For higher education, we offer four concrete pedagogical implications. (i) Scaffolded-explanation templates for diagnosis-and-explanation queries. When the retrieval-use type is diagnosis-and-explanation (Section 4.3), the L4 policy should not return the corrected code as the primary output. It should return a structured explanation that names the error, restates the relevant concept in plain language, points to the course material at L3 where the concept is taught, and asks the learner to attempt the fix before requesting the corrected code. This is a direct application of the cognitive-apprenticeship sequence of modeling, coaching, and fading (Yin and Yin, 2024; Liao et al., 2024), with the L4 template providing the modeling step. (ii) Practice-item retrieval as a forethought-phase prompt. Practice-item queries are the most strongly augmentation-oriented in our corpus (98.8% of such queries), so the L4 template can prompt the learner to articulate which concept they want to practice on and what they already understand about it, before returning a practice item. This aligns the interaction with the forethought phase of Zimmerman’s self-regulated learning cycle as operationalized in the chatbot design review by Wu et al. (2025). (iii) Reflection prompts at session end. Because long sessions with high LAI variability may signal a transition from augmentation to automation, the L4 policy can append a brief reflection prompt at the end of a session of more than n queries, asking the learner to summarize what they learned in their own words. This implements the self-reflection phase of self-regulated learning and is designed to mitigate the metacognitive-laziness effect identified by Fan et al. (2025). (iv) Course-team triage by category. Mean LAI per category, together with within-category augmentation share, can be used by the course team between cohorts to identify topics where students disproportionately ask automation-style questions. These are candidate topics for redesigned assessments or in-class formative activities.

Each of these instructional implications is a hypothesis grounded in the behavioral patterns we observe, not a tested recommendation. The next study, which would pair the L4 policy variants with assessment outcomes, is the one that can test them.

## 11 Limitations

Several limitations of this study should be reported explicitly.

Small original sample with substantial non-response bias. The 12,330 queries originate from 34 students who consented to participate in the original deployment study, which is 22% of the 154 enrolled students (Thway et al., 2025). Non-respondents may differ systematically from respondents in their AI use, their study habits, and the kinds of queries they would have generated. The corpus is a complete enumeration of one self-selected subgroup’s queries, not a sample of the cohort. The non-response bias also affects the generalizability of the survey-versus-behavior comparison in Section 8.2.

Clustering structure. The corpus is clustered at the student level. The public release does not include a consistent student identifier throughout the entire log, so we cannot fit a multilevel logistic model with student as a random effect. The Cramer’s V and chi-square statistics we report should be read as descriptive summaries of contingency tables in this corpus, not as inferential tests about an underlying population. Future deployments that release de-identified yet consistent participant identifiers would allow a within-student behavioral analysis.

Model-version effect. The chatbot used Anthropic Claude 3 (Sonnet) at the time of data collection. That model is no longer current in the Claude family. Response style, conciseness, and willingness to push back on automation-style requests may vary across subsequent models, which could in turn shape the kinds of queries students choose to submit. We cannot estimate the magnitude of this effect from the public dataset. We name it here as a threat to the temporal validity of the descriptive patterns.

One deployment, one course. The corpus comes from a single semester of a single undergraduate data science module. The category distribution, the LAI mean, and the predictive AUC are all conditional on that course, that lecturer, and that cohort.

Rule-based labeling. Both the topical categorization and the augmentation label are produced by hand-written rules. This makes them auditable but also encodes our prior view of what augmentation looks like, and the audit reported in Section 4.3 makes the cost of this design choice explicit. A natural next step is to derive categories statistically (for example, via latent Dirichlet allocation or structural topic modeling over the cleaned corpus) and to compare the resulting category structure against the rule-based one. We did not adopt that approach in the present study because the priority was a fully auditable and reproducible coding scheme.

Query level, not learning level. We measure what the learner asked for, not what they learned, understood, or retained. This limitation is fundamental: the public dataset contains no assessment performance, no pre- or post-tests, no think-aloud transcripts, no dialogue history, and no measure of conceptual gain. Claims about augmentation orientation concern the surface form of a single query, not about cognitive engagement, metacognitive activity, or self-regulated learning. We frame the contribution as a behavioral analytics framework rather than as a measurement of learning. Linking this kind of log to assessment outcomes is the next required study, not an extension.

LAI is heuristic, not validated. The LAI weights (alpha, beta, gamma, delta, lambda) are chosen *a priori* rather than learned. The five components of the LAI are derived from the same rule-based signals that produce the augmentation label, creating a circularity: the index does not measure an independent construct; it weights and scales the labeling signal. The reported AUC of 0.792 reflects internal consistency with the labeling rules, not external predictive validity against an independent outcome. A sensitivity analysis (each weight perturbed by plus or minus 0.10 in turn) leaves the top-four LAI category ranking unchanged in this corpus, although absolute LAI values would shift. External validation against grade outcomes or a learning-gain measure is the only way to test whether the LAI tracks anything beyond its own definition.

Single-class topical labels. A query about plotting a regression’s residuals is genuinely about both visualization and modeling, and the priority-ordered scheme assigns it to one category, which slightly undercounts cross-cutting queries in either.

## 12 Conclusion

We took the 12,330-query log of a public, course-specific RAG chatbot, a corpus generated by 34 consenting students in a single 14-week deployment of Prof. Leodar (Thway et al., 2025). We asked one descriptive question: in observable behavioral terms, what is the chatbot being asked to do for the student? Within this corpus, 46.6% of interactions are classified by our rule-based scheme as requests to scaffold reasoning, and 53.4% as requests for a finished answer. The augmentation share is highest in the technical categories, where students of an introductory data science course need the most help. The most reliable behavioral predictor of augmentation orientation is the retrieval-use type implied by the query, rather than the topical category (Cramer’s V 0.226 versus 0.166). A heuristic LAI can rank categories within this corpus as an instructor would. Our contribution is not a judgment that this chatbot is good or bad for learning, nor a claim that augmentation-oriented queries produce better learning outcomes, because the data do not support either claim. The contribution is a transparent and reproducible behavioral analytics framework for course-specific RAG. Linking this kind of analysis to assessment data, ideally in a deployment that also releases student-level identifiers and an updated language-model backend, is the natural next step in the study and can test whether the patterns we describe carry educational weight.

## Data Availability

The datasets presented in this study can be found in online repositories. The names of the repository/repositories and accession number(s) can be found in the article/supplementary material.

## References

[ref1] Albadarin Y. Saqr M. Pope N. Tukiainen M. (2024). A systematic literature review of empirical research on ChatGPT in education. Discov. Educ. 3:60. doi: 10.1007/s44217-024-00138-2

[ref2] Barnett S. Kurniawan S. Thudumu S. Brannelly Z. Abdelrazek M.. Seven failure points when engineering a retrieval augmented generation system. Proceedings of the IEEE/ACM 3rd International Conference on AI Engineering: Software Engineering for AI. (2024):194–199. doi: 10.1145/3644815.3644945.

[ref3] Batista J. Mesquita A. Carnaz G. (2024). Generative AI and higher education: trends, challenges, and future directions from a systematic literature review. Information 15:676. doi: 10.3390/info15110676

[ref4] Chan C. K. Y. Hu W. (2023). Students’ voices on generative AI: perceptions, benefits, and challenges in higher education. Int. J. Educ. Technol. High. Educ. 20:43. doi: 10.1186/s41239-023-00411-8

[ref5] Chen J. Lin H. Han X. Sun L.. Benchmarking large language models in retrieval-augmented generation. Proceedings of the AAAI Conference on Artificial Intelligence. (2024). 38:17754–17762. doi: 10.1609/aaai.v38i16.29728.

[ref6] Cotton D. R. E. Cotton P. A. Shipway J. R. (2024). Chatting and cheating: ensuring academic integrity in the era of ChatGPT. Innov. Educ. Teach. Int. 61, 228–239. doi: 10.1080/14703297.2023.2190148

[ref7] Crompton H. Burke D. (2023). Artificial intelligence in higher education: the state of the field. Int. J. Educ. Technol. High. Educ. 20:22. doi: 10.1186/s41239-023-00392-8

[ref8] Dwivedi Y. K. Kshetri N. Hughes L. Slade E. L. Jeyaraj A. Kar A. K. . (2023). So what if ChatGPT wrote it? Multidisciplinary perspectives on opportunities, challenges and implications of generative conversational AI. Int. J. Inf. Manag. 71:102642. doi: 10.1016/j.ijinfomgt.2023.102642

[ref9] Fan Y. Tang L. Le H. Shen K. Tan S. Zhao Y. . (2025). Beware of metacognitive laziness: effects of generative artificial intelligence on learning motivation, processes, and performance. Br. J. Educ. Technol. 56, 489–530. doi: 10.1111/bjet.13544

[ref10] Gao Y. Xiong Y. Gao X. Jia K. Pan J. Bi Y. . (2024). Retrieval-augmented generation for large language models: a survey. arXiv preprint arXiv:2312.10997. doi: 10.48550/arXiv.2312.10997

[ref11] Huang Y. Huang J. X. (2024). A survey on retrieval-augmented text generation for large language models. arXiv preprint arXiv:2404.10981. doi: 10.48550/arXiv.2404.10981

[ref12] Izacard G. Lewis P. Lomeli M. Hosseini L. Petroni F. Schick T. . (2023). Atlas: few-shot learning with retrieval augmented language models. J. Mach. Learn. Res. 24, 1–43.

[ref13] Jeong S. Baek J. Cho S. Hwang S. J. Park J. C. Adaptive-RAG: learning to adapt retrieval-augmented large language models through question complexity. Proceedings of the 2024 Conference of the North American Chapter of the Association for Computational Linguistics (NAACL). (2024):7036–7050. doi: 10.18653/v1/2024.naacl-long.389.

[ref14] Jiang Z. Xu F. Gao L. Sun Z. Liu Q. Dwivedi-Yu J. . Active retrieval augmented generation. Proceedings of the 2023 Conference on Empirical Methods in Natural Language Processing (EMNLP). (2023) 7969–7992. doi: 10.18653/v1/2023.emnlp-main.495.

[ref15] Jo H. (2024). From concerns to benefits: a comprehensive study of ChatGPT usage in education. Int. J. Educ. Technol. High. Educ. 21:35. doi: 10.1186/s41239-024-00471-4

[ref16] Kasneci E. Sessler K. Kuchemann S. Bannert M. Dementieva D. Fischer F. . (2023). ChatGPT for good? On opportunities and challenges of large language models for education. Learn. Individ. Differ. 103:102274. doi: 10.1016/j.lindif.2023.102274

[ref17] Lee D. Arnold M. Srivastava A. Plastow K. Strelan P. Ploeckl F. . (2024). The impact of generative AI on higher education learning and teaching: a study of educators’ perspectives. Comp. Educ. 6:100221. doi: 10.1016/j.caeai.2024.100221, 38826717

[ref18] Lewis P. Perez E. Piktus A. Petroni F. Karpukhin V. Goyal N. . (2020). Retrieval-augmented generation for knowledge-intensive NLP tasks. Adv. Neural Inform. Process. Syst. 33, 9459–9474.

[ref19] Li Z. Wang Z. Wang W. Hung K. Xie H. Wang F. L. (2025). Retrieval-augmented generation for educational application: a systematic survey. Comp. Educ. 8:100417. doi: 10.1016/j.caeai.2025.100417, 38826717

[ref20] Liao J. Zhong L. Zhe L. Xu H. Liu M. Xie T. (2024). Scaffolding computational thinking with ChatGPT. IEEE Trans. Learn. Technol. 17, 1628–1642. doi: 10.1109/TLT.2024.3392896

[ref21] Lo C. K. (2023). What is the impact of ChatGPT on education? A rapid review of the literature. Educ. Sci. 13:410. doi: 10.3390/educsci13040410

[ref22] Mialon G. Dessi R. Lomeli M. Nalmpantis C. Pasunuru R. Raileanu R. . (2023). Augmented language models: a survey. Trans. Machine Learn. Res.

[ref23] Nemeth R. Tatrai A. Szabo M. Zaletnyik P. T. Tamasi A. (2025). Exploring the use of retrieval-augmented generation models in higher education: a pilot study on artificial intelligence-based tutoring. Soc. Sci. Humanit. Open 12:101751. doi: 10.1016/j.ssaho.2025.101751

[ref24] Rudolph J. Tan S. Tan S. (2023). ChatGPT: bullshit spewer or the end of traditional assessments in higher education? J. Appl. Learn. Teach. 6, 342–363. doi: 10.37074/jalt.2023.6.1.9, 42219738

[ref25] Saude S. Barros D. Almeida I. (2024). Impacts of generative artificial intelligence in higher education: research trends and students’ perceptions. Soc. Sci. 13:410. doi: 10.3390/socsci13080410

[ref26] Shi Y. Yu K. Dong Y. Chen F. (2025). Large language models in education: a systematic review of empirical applications, benefits, and challenges. Comput. Educ. Artif. Intell. 10:100529. doi: 10.1016/j.caeai.2025.100529

[ref27] Su J. Yang W. (2023). Unlocking the power of ChatGPT: a framework for applying generative AI in education. ECNU Rev. Educ. 6, 355–366. doi: 10.1177/20965311231168423

[ref28] Swacha J. Gracel M. (2025). Retrieval-augmented generation (RAG) chatbots for education: a survey of applications. Appl. Sci. 15:4234. doi: 10.3390/app15084234

[ref29] Thway M. Recatala-Gomez J. Lim F. S. Hippalgaonkar K. Ng L. W. T. (2025). Harnessing GenAI for higher education: a study of a retrieval augmented generation chatbot’s impact on learning. J. Chem. Educ. 102, 3849–3860. doi: 10.1021/acs.jchemed.5c00113

[ref30] Tlili A. Shehata B. Adarkwah M. A. Bozkurt A. Hickey D. T. Huang R. . (2023). What if the devil is my guardian angel: ChatGPT as a case study of using chatbots in education. Smart Learn. Environ. 10:15. doi: 10.1186/s40561-023-00237-x

[ref31] Trivedi H. Balasubramanian N. Khot T. Sabharwal A. Interleaving retrieval with chain-of-thought reasoning for knowledge-intensive multi-step questions. Proceedings of the 61st Annual Meeting of the Association for Computational Linguistics (ACL) (2023) 10014–10037. doi: 10.18653/v1/2023.acl-long.557.

[ref32] Wei J. Wang X. Schuurmans D. Bosma M. Ichter B. Xia F. . (2022). Chain-of-thought prompting elicits reasoning in large language models. Adv. Neural Inform. Process. Syst. 35, 24824–24837.

[ref33] Wu X. Y. Radloff J. D. Yeter I. H. Wang L. Chiu T. K. F. (2025). Designing artificial intelligence chatbots for self-regulated learning from a systematic review based on Habermas’s three interests. Interact. Learn. Environ. doi: 10.1080/10494820.2025.2563086

[ref34] Yan L. Sha L. Zhao L. Li Y. Martinez-Maldonado R. Chen G. . (2024). Practical and ethical challenges of large language models in education: a systematic scoping review. Br. J. Educ. Technol. 55, 90–112. doi: 10.1111/bjet.13370

[ref35] Yan S. Yu D. Yang H. Yue X. Lin W. (2024). Corrective retrieval augmented generation. arXiv preprint arXiv:2401.15884. doi: 10.48550/arXiv.2401.15884

[ref36] Yin D. S. Yin X. (2024). Scaffolding learning: from specific to generic with large language models. PLoS One 19:e0310409. doi: 10.1371/journal.pone.0310409, 39302920 PMC11414939

